# Stress-induced immunosuppression affecting immune response to Newcastle disease virus vaccine through “miR-155-CTLA-4” pathway in chickens

**DOI:** 10.7717/peerj.14529

**Published:** 2023-02-27

**Authors:** Jie Wen, Yiru Wu, Jianwei Han, Yufei Tian, Chaolai Man

**Affiliations:** Harbin Normal University, Harbin, China

**Keywords:** Chicken, MiR-155, *CTLA-4* gene, Stress-induced immunosuppression, Newcastle disease virus

## Abstract

MiR-155 and CTLA-4 are important factors involved in the regulation of immune function. However, there is no report about their involvement in function regulation of stress-induced immunosuppression affecting immune response. In this study, the chicken model of stress-induced immunosuppression affecting immune response (simulation with dexamethasone and immunization with Newcastle disease virus (NDV) attenuated vaccine) was established, then the expression characteristics of miR-155 and *CTLA-4* gene were analyzed at several key time points during the processes of stress-induced immunosuppression affecting NDV vaccine immune response at serum and tissue levels. The results showed that miR-155 and *CTLA-4* were the key factors involved in stress-induced immunosuppression and NDV immune response, whose functions involved in the regulation of immune function were different in different tissues and time points, and 2 day post immunization (dpi), 5dpi and 21dpi were the possible key regulatory time points. *CTLA-4*, the target gene of miR-155, had significant game regulation relationships between them in various tissues, such as bursa of *Fabricius*, thymus and liver, indicating that miR-155-CTLA-4 pathway was one of the main mechanisms of their involvement in the regulations of stress-induced immunosuppression affecting NDV immune response. This study can lay the foundation for in-depth exploration of miR-155-CTLA-4 pathway involved in the regulation of immune function.

## Introduction

Stress-induced immunosuppression is one of the most common problems in intensive poultry production. Many factors (such as too cold, overheating, overcrowding, changing feeds, noise, transfer, *etc.*) can cause immunosuppression, which often leads to poor or even failure of vaccine immunization, resulting in enormous economic losses to poultry industry. If we can comprehensively understand the molecular mechanism of stress-induced immunosuppression, it can provide directional theoretical guidance for solving this problem. MiR-155, a key regulator of immune function, plays an indispensable regulatory role in the processes of activation, proliferation, differentiation of immune cells and antibody production (Lind & Ohashi, 2014; Chen et al., 2021; Seddiki et al., 2014). Moreover, stress can induce expression changes of miR-155 in B cells (Sun et al., 2019) and T cells (Daniel et al., 2018), indicating that miR-155 may be an important regulator involved in immunosuppression.

The cytotoxic T-lymphocyte-associated antigen 4 (*CTLA-4*), the key immune checkpoint and target gene of miR-155, plays a key regulatory role in mediating self-tolerance, preventing autoimmunity, and protecting tissues from immune attack (Zhang & Zheng, 2020; Chikuma, 2017; Chao et al., 2019). The *CTLA-4* gene is up-regulated in activated CD4^+^ and CD8^+^ T cells, and plays an important role in anti-tumor immunity through blocking co-stimulatory signals and inhibiting the activation and proliferation of T cells (Chan et al., 2014; Krummey & Ford, 2014; Rotte, 2019; Van Coillie, Wiernicki & Xu, 2020). Interestingly, immunosuppressive infectious bursa fabricius virus (IBDV) infecting chicken can induce changes in the expression of *CTLA-4* in bursa of *Fabricius*, spleen and cecal tonsil (Ruan et al., 2020), and glucocorticoid can up-regulate the expression of *CTLA-4* gene in activated T cell (Xia, Gasser & Feige, 1999), suggesting that CTLA-4 may play a vital role in the process of stress-induced immunosuppression. However, there are still no reports on the functions and characteristics of miR-155-CTLA-4 axis in stress-induced immunosuppression affecting immune response.

In order to further explore the roles and properties of miR-155-CTLA-4 pathway in immune regulation, the dynamic changes of serum circulating and tissue miR-155 and *CTLA-4* were identified at different time points during the processes of immunosuppression simulated by dexamethasone and its influence on immune response induced by attenuated vaccine of the Newcastle disease virus (NDV), respectively. In addition, the dynamic game relationships between miR-155 and *CTLA-4* gene were analyzed in different tissues during these processes. Studying the function and characteristics of miR-155-CTLA-4 axis involved in stress-induced immunosuppression affecting immune response has theoretical and practical research value for in-depth understanding of the molecular mechanism of immune function regulation and the diagnosis of immune response effects.

## Materials and methods

### Ethics statement

The proposed study protocol was supported by the Institutional Animal Care and Use Committee (IACUC) of the Harbin Normal University (HNUARIA2021001). All animals were handled in compliance with the guidelines set forth by the Guide for the Care and Use of Laboratory Animals and according to the guidelines of the Institutional Animal Care and Use Committee.

### Experimental grouping and sample collection

Two hundred non-immunized one-day-old Hy-Line Brown chickens were obtained from Xiangfang farm in Harbin city and equally divided into 4 groups: control group, Dex group, ND group and Dex+ND group. Adequate feed and drinking water were offered ad libitum, and different treatment groups were reared in isolation. At 7-day-old, sterile water containing dexamethasone (Dex) (1.5 mg/L, Shanxi Ruicheng Kelong Veterinary Pharmaceutical Co., Ltd., China) was free to drink in Dex group and Dex +ND group for five consecutive days. At 12-day-old, chickens of ND group and Dex+ND group were administered with NDV LaSota vaccine (Harbin veterinary research institute, China) by eye drop (about 25 µL) according to the instruction, and the chickens from control group and Dex group were treated with NDV vaccine diluent (Harbin veterinary research institute, China). Several tissues including heart, liver, spleen, bursa of *Fabricius*, thymus, glandular stomach, cecal tonsil and serums of three chickens were collected on 1day post immunization (dpi), 2dpi, 3dpi, 4dpi, 5dpi, 7dpi, 14dpi, 21dpi, 28dpi, and 35dpi. Three randomly-selected chickens from each group were humanely euthanized by an anesthetic overdose of Sumianxin II (0.2 mL/kg body weight; Shengda Animal Medicine Co., Ltd., Dunhua, China) at each time point. All samples were frozen in liquid nitrogen and stored at −80 °C.

### Serum antibody detection

The antibody titers of chicken serum from ND group were determined by hemagglutination (HA) and hemagglutination inhibition (HI) experiments with standard antigen of LaSota strain from Harbin Veterinary Research Institute of China. HA and HI assays were mainly followed by the method of Chen et al. (2011). Body, thymus, spleen and bursa of *Fabricius* of three chickens from each group were weighed at each time point. Organ coefficients were calculated according to the formula: organ coefficient = tissue weight (g)/body weight (g) ×100%.

### Expression level analysis of miR-155 and *CTLA-4*

Total RNA was extracted with TRIzol (Invitrogen, US) from each tissue sample followed by the instructions. 300 ng RNA of each sample was used to perform reverse transcription reaction using the reverse transcription kit FSQ-301 (TOYOBO, Shanghai, China) according to the instructions. The reverse transcription primers were 5′-CTCAACTGGTGTCGTGGAGTCGGCAATTCAGTTGAGCCCCTATC-3′ (miR-155), oligo(dT) (*CTLA-4* gene and internal reference *β-actin*) and 5′-AACGCTTCACGAATTTGCGT-3′ (internal reference *U6*), respectively. Quantitative real-time PCR (qRT-PCR) was used to analyze the expression levels of different targets. The qRT-PCR primers were as follows: miR-155: 5′-ACACTCCAGCTGGGTTAATGCTAATCGTGA-3′ and 5′-TGGTGTCGTGGAGTCG-3′; *U6*: 5′-CTCGCTTCGGCAGCACA-3′ and 5′-AACGCTTCACGAATTTGCGT-3′; *CTLA-4*: 5′-TGCCGAAGTAATGGAAGTGA-3′and 5′-TTTCAGTGAACTTGTCGCCT-3′; *β-actin*: 5′-TATGTGCAAGGCCGGTTTC-3′ and 5′-CACCAACGTAGCTGTCTTTCTG-3′. All qRT-PCR systems were 10 µL including: 5  µL  1 ×SYBR Green I (TOYOBO, Shanghai, China), 0.2  µL  50 ×ROX reference dye (TOYOBO, Shanghai), 0.3  µL of each primer, 1.2 µL cDNA and 3  µL ddH_2_O. QRT-PCR processes were: 95 °C/1 min; 45 cycles of 95 °C/15s, 60 °C/30s, 72 °C/30s. The qRT-PCR system doses and reaction procedures of *U6, β-actin* and *CTLA-4* were the same as miR-155.

### Statistical analysis

The relative expression levels of miR-155 and *CTLA-4* in serum and tissues were calculated using 2^−ΔΔCt^ method. The data were analyzed using SPSS 20.0 software (independent sample *t*-test and one-way analysis of variance) and graphed by GraphPad Prism 8.0 software. All experiments were repeated three times.

## Results and Discussion

### The identification of immunosuppressed chicken models

**Figure 1:**
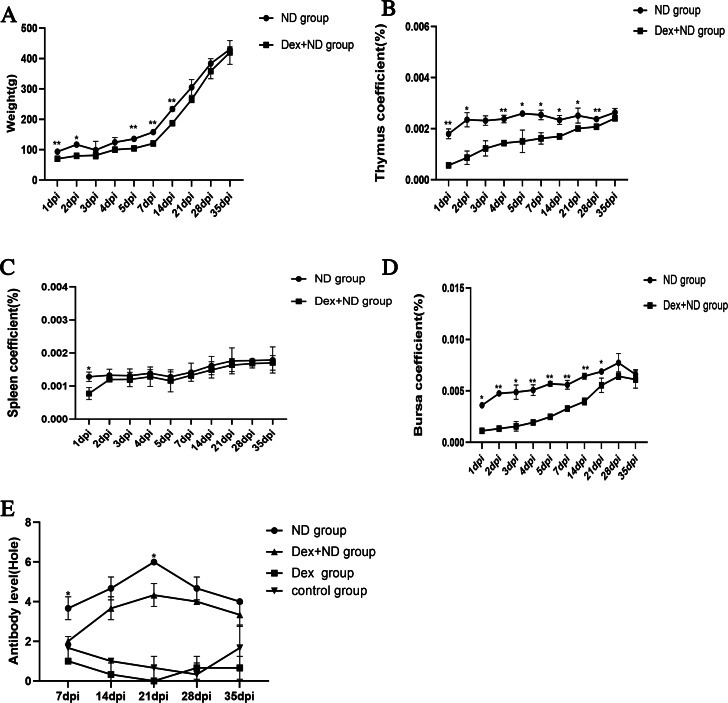
Identification of Dex-induced immunosuppressed chicken models. (A, B, C, D) Body weights and organ coefficients of thymus, spleen and bursa in ND group and Dex+ND group, respectively; (E) NDV antibody levels in control group, Dex group, ND group and Dex+ND group. An asterisk (*) represents statistically significant between different groups at the same time point (*P* < 0.05), two asterisks (**) represent extremely statistically significant between different groups at the same time point (*P* < 0.01).

Dexamethasone is one of the classic methods for simulating and preparing stress-induced immunosuppressed chicken (Zhai et al., 2020; Guo et al., 2020; Su et al., 2021). The identification results of immunosuppressed chicken models showed that the body weights and organ coefficients of thymus, spleen and bursa of Dex+ND group were significantly lower than that of ND group from 1dpi to 35dpi (Figs. 1A–1D). HI results showed that the antibody levels of ND group and Dex+ND group gradually increased after immunization, all reached the peaks on 21dpi, and then slowly decreased. However, ND group antibody levels were significantly higher than Dex+ND group. No corresponding antibodies were detected in the control group and Dex group (Fig. 1E). The above results showed that the Dex-induced immunosuppressed chicken models were established successfully.

### Expression analysis of miR-155 and *CTLA-4* in Dex-induced immunosuppressed chicken

**Figure 2:**
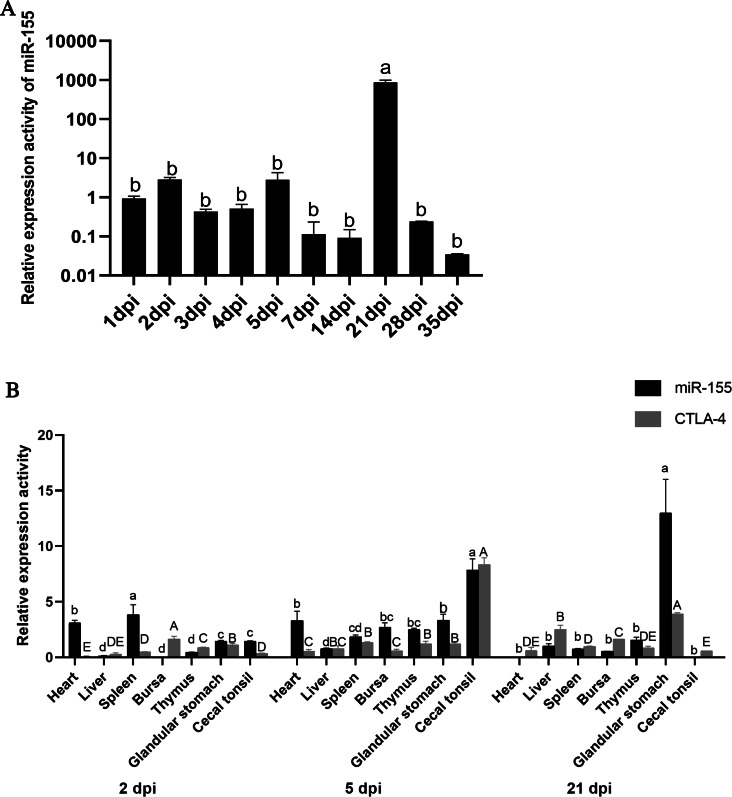
Expression levels of miR-155 and *CTLA-4* in Dex group. (A) Expression levels of serum circulating miR-155 in Dex group. (B) Expression levels of tissue miR-155 and *CTLA-4* in Dex group, respectively. The expression levels of miR-155 and *CTLA-4* were calculated with 2^−ΔΔCt^ method between the Dex group and control group. The different letters on columns represented significant differences (*P* < 0.05), while the same letters on columns represented no significant differences (*P* > 0.05). The lowercase letters of (A) represent the significances of miR-155 at different time points in Dex group. The lowercase letters of (B) represent the significant differences of miR-155 between different tissues at the same time point. The uppercase letters of (B) represent the significant differences of *CTLA-4* gene between different tissues at the same time point.

Studying serum circulating miR-155 expression levels in different stages of Dex-induced immunosuppression can provide insight into the roles and properties of circulating miR-155. For the convenience of expression, the processing time points of the four groups were marked with the same method (dpi). In Dex group, serum circulating miR-155 showed up-regulation trend during 1∼2dpi, and then decreased from 2dpi to 4dpi. On 5dpi, circulating miR-155 increased significantly (*P* < 0.05). During 7dpi∼21dpi, serum circulating miR-155 only increased significantly on 21dpi (*P* < 0.05), but decreased at other time points (Fig. 2A). The above results indicated Dex had a significant effect on the expression levels of serum circulating miR-155, and 2dpi, 5dpi and 21dpi were the possible key time points for their significant upregulations, which provided the candidate time points for stress-induced immunosuppression assays.

In order to further analyze the possible tissue sources and functional characteristics of circulating miR-155 at the above critical time points, the expression changes of tissue miR-155 and its target gene *CTLA-4* were analyzed in seven tissues (heart, liver, spleen, bursa of *Fabricius*, thymus, glandular stomach, caecal tonsil), respectively. We artificially set up three expression levels: over two for high expression, less 1 for low expression, and medium expression between 1 and 2. QRT-PCR results showed that the expression activities of miR-155 in different tissues at the same time points and the same tissues at different time points were all significantly different (*P* < 0.05), indicating that miR-155 was a key regulator involved in Dex-induced immunosuppression (Fig. 2B). For example, on 2dpi, miR-155 was highly expressed in heart and spleen, suggesting that the up-regulation of serum circulating miR-155 might be associated to the heart and spleen; on 5dpi, miR-155 was expressed in heart, bursa of *Fabricius*, thymus, glandular stomach, and cecal tonsils at moderate or high levels, suggesting that the up-regulation of circulating miR-155 might be related to these tissues; on 21dpi, miR-155 was only highly expressed in glandular stomach, suggesting that the significant up-regulation of serum circulating miR-155 might be mainly related to it. Overall, miR-155 showed upward trends in liver and glandular stomach among the three time points, but downward trend in spleen. And the expression activities of miR-155 in heart, bursa of *Fabricius*, thymus and cecal tonsil reached the peak value on 5dpi, suggesting that the functions of miR-155 involved in Dex-induced immunosuppression were different in different tissues and time points.

In addition, qRT-PCR results showed that the expression activities of *CTLA-4* at the same time point in different tissues and the same tissue at different time points were also significantly different (*P* < 0.05), indicating that *CTLA-4* was a key gene participating in Dex-induced immunosuppression (Fig. 2B). For example, on 2dpi, *CTLA-4* was moderately expressed in bursa of *Fabricius* and glandular stomachs, and lowly expressed in other tissues; on 5dpi, *CTLA-4* was highly expressed in cecal tonsil, moderately expressed in spleen, thymus and glandular stomach, and lowly expressed in the other tissues; on 21dpi, *CTLA-4* was highly expressed in liver and glandular stomach, moderately expressed in bursa of *Fabricius*, and lowly expressed in the other tissues. On the whole, *CTLA-4* showed upward trends in the liver, and glandular stomach among the three time points, and valley values in the bursa of *Fabricius* on 5dpi, while reached peak value in the spleen, thymus and cecal tonsils on 5dpi, suggesting that the functions of *CTLA-4* involved in stress-induced immunosuppression were also different in different tissues and time points.

In brief, our research results fully showed that both miR-155 and *CTLA-4* were key factors involved in Dex-induced immunosuppression. Moreover, *CTLA-4* was the target gene of miR-155 (Zhang et al., 2017), the miR-155-CTLA-4 pathway might play an important role in the process of Dex-induced immunosuppression. For example, according to the relative expression activities of miR-155 and *CTLA-4*, there were obvious game regulation relationships between miR-155 and *CTLA-4* in heart, spleen, bursa of *Fabricius* and cecal tonsil on 2dpi; heart and bursa of *Fabricius* on 5dpi, and bursa of *Fabricius* and thymus on 21dpi. Of course, other regulatory pathways might also exist for miR-155 and *CTLA-4* in cells, so the specific mechanisms needed to be further studied in the future.

### The role and properties of miR-155 and *CTLA-4* in the process of Dex affecting NDV immune response

Since the above results proved that the miR-155-CTLA-4 pathway was one of the key mechanisms involved in Dex-induced immunosuppression, it remained unknown whether this mechanism was the main pathway that immunosuppression affected immune response. In order to further explore whether miR-155-CTLA-4 axis played a key regulatory role in Dex-induced immunosuppression affecting the immune response to NDV vaccine, the expression changes of serum circulating miR-155 between ND group and Dex+ND group were analyzed firstly, then the expression characteristics of tissue miR-155 and *CTLA-4* were identified. The expression level ranges were still in the same way as the Dex group.

**Figure 3:**
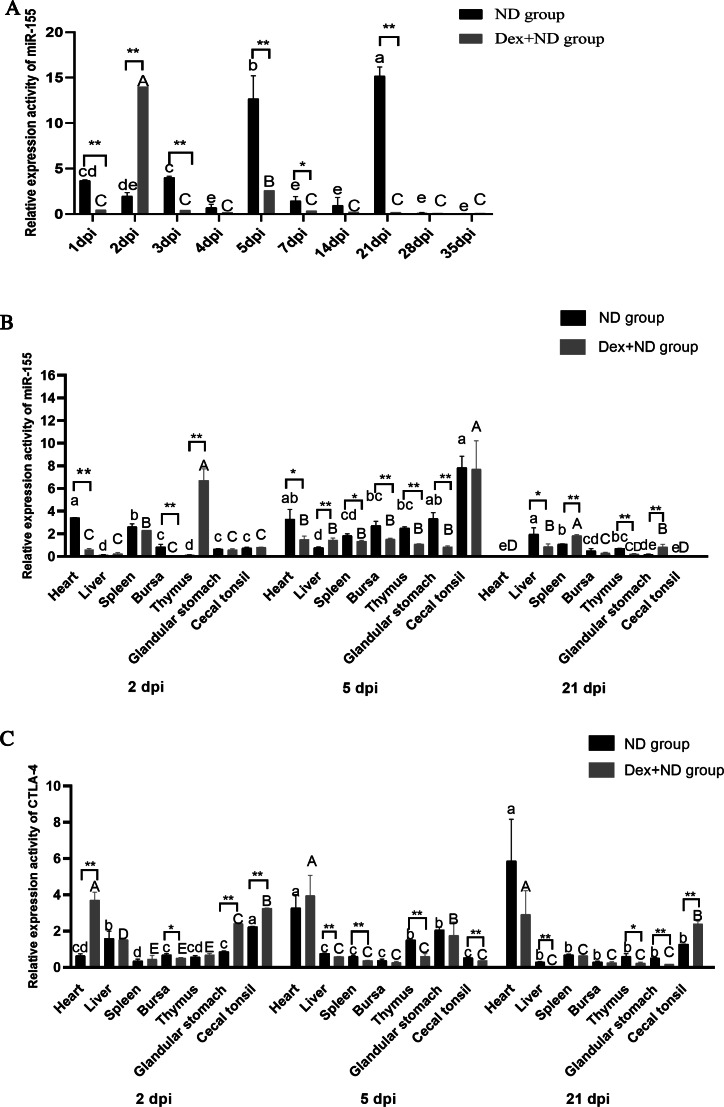
The expression levels of miR-155 and *CTLA-4* in ND group and Dex+ND group. (A) The expression levels of serum circulating miR-155 in ND group and Dex+ND group. (B) The expression levels of tissue miR-155 in ND group and Dex+ND group. (C) The expression levels of tissue *CTLA-4* gene in ND group and Dex+ND group. The expression levels of miR-155 and *CTLA-4* were calculated with 2^−ΔΔCt^ method between treatment group (ND group or Dex+ND group) and control group. The different letters on columns represented significant differences (*P* < 0.05), while the same letters on columns represented no significant differences (*P* > 0.05). The lowercase letters of (A) represented the significant differences of miR-155 at different time points in ND group. The uppercase letters of (A) represented the significant differences of miR-155 at different time points in Dex+ND group. The lowercase letters of (B and C) represented the significant differences of miR-155 and *CTLA-4* of ND group between different tissues at the same time point, respectively. The uppercase letters of (B and C) represented the significant differences of miR-155 and *CTLA-4* of Dex+ND group between different tissues at the same time point, respectively. An asterisk (*) represents statistically significant differences of the same tissue between different ND group and Dex+ND group (*P* < 0.05), two asterisks (**) represent extremely statistically significant differences of the same tissue between ND group and Dex+ND group (*P* < 0.01).

QRT-PCR results showed that serum circulating miR-155 of ND group was highly expressed on 1dpi, 3dpi, 5dpi and 21dpi (*P* < 0.05), moderately expressed on 2dpi and 7dpi (*P* < 0.05), lowly expressed at other time points. However, the serum circulating miR-155 of Dex+ND group was highly expressed only on 2dpi and 5dpi (*P* < 0.05), and lowly expressed at other time points (Fig. 3A). There were significant differences in the expression pattern of circulating miR-155 between the ND group and Dex+ND group, indicating that serum circulating miR-155 was a key regulator involved in the process of Dex affecting NDV immune response. It was worth mentioning that the expression levels of circulating miR-155 were significantly different between ND group and Dex+ND group within 3 days after immunization, suggesting that circulating miR-155 could be used as a potential molecular marker for early diagnosis of NDV vaccine immunization effect.

We still selected 2dpi (innate immunity stage), 5dpi (transition stage from innate immunity to specific immunity) (Cao, Xiong & Zhi, 2013) and 21dpi (specific immunity stage) as possible key time points for in-depth study, because there were significant differences between ND group and Dex+ND group. The qRT-PCR analysis of tissue miR-155 expression activities between ND group and Dex+ND group found that there were significant differences between different tissues at the same time points and the same tissues at different time points (*P* < 0.05) (Fig. 3B), further indicating that miR-155 was a key regulator involved in the immune response induced by NDV vaccine and Dex affecting NDV immune response. In comparison with the ND group, the expression activities of tissue miR-155 in heart (down-regulation) (*P* < 0.01), bursa of *Fabricius* (down-regulation) (*P* < 0.01) and thymus (up-regulation) (*P* < 0.01) were significantly different in Dex+ND group on 2dpi, suggesting that miR-155 participated in the innate immune response of these tissues during Dex affecting NDV vaccine response; on 5dpi, the expression activities of miR-155 in heart (down-regulation) (*P* < 0.05), spleen (down-regulation) (*P* < 0.05), liver (up-regulation) (*P* < 0.01), bursa of *Fabricius* (down-regulation) (*P* < 0.01), glandular stomach (down-regulation) (*P* < 0.01) and thymus (down-regulation) (*P* < 0.01) had significant differences. It was suggested that miR-155 was mainly involved in the initial stage of Dex affecting NDV vaccine specific immune response in these tissues; on 21dpi, there were significant differences in the expression activities of miR-155 in liver (down-regulation) (*P* < 0.05), spleen (up-regulation) (*P* < 0.01), thymus (down-regulation) (*P* < 0.01) and glandular stomach (up-regulation) (*P* < 0.01). It was suggested that miR-155 was also involved in the specific immunity stage of Dex affecting NDV vaccine in these tissues. In summary, the tissue miR-155 of the two groups had extremely significant changes in multiple tissues on 2dpi, suggesting that 2dpi was a critical time point for Dex affecting NDV innate immune response; on 5dpi, the expression activities of most candidate tissues showed an upward trend over medium levels, indicating that 5dpi was a key time point for tissue miR-155 participating in the initiation of specific immune response; on 21dpi, tissue miR-155 was lowly expressed in most candidate tissues, and the number of significant differences between the two groups decreased, suggesting that the ability of Dex affecting NDV specific immune response through miR-155 decreased. Interestingly, in Dex+ND group, the expression activities of miR-155 in thymus of was down-regulated from 2dpi to 21dpi, but up-regulated to the peak in the heart, liver, bursa of *Fabricius*, glandular stomach, and cecal tonsils on 5 dpi, indicating that 5 dpi was the most critical time point when miR-155 participated in the process of Dex affecting NDV immune response.

QRT-PCR results showed that the expression activities of *CTLA-4* in different tissues at the same time points and the same tissues at different time points were also significantly different (*P* < 0.05) (Fig. 3C), further indicating that *CTLA-4* was also a key factor of Dex affecting NDV immune response. In comparison with ND group, on 2dpi, the expression activities of *CTLA-4* in heart (down-regulation) (*P* < 0.01), bursa of *Fabricius* (up-regulation) (*P* < 0.01) and glandular stomach (down-regulation) (*P* < 0.01) were significantly different in Dex+ND group, suggesting that *CTLA-4* was involved in the Dex influencing NDV innate immune response process through these tissues; on 5dpi, there were significant differences in the expression activities of *CTLA-4* in the liver (up-regulation) (*P* < 0.01), spleen (up-regulation) (*P* < 0.01), thymus (up-regulation) (*P* < 0.05) and cecal tonsils (up-regulation) (*P* < 0.01), suggesting that *CTLA-4* was also mainly involved in the initial stage of Dex affecting NDV specific immune response through these tissues; on 21dpi, there were also significant differences in the expression activities of *CTLA-4* in liver (up-regulation) (*P* < 0.01), thymus (up-regulation) (*P* < 0.05), glandular stomach (up-regulation) (*P* < 0.01) and cecal tonsil (down-regulation) (*P* < 0.01). It suggested that *CTLA-4* was involved in Dex influencing NDV specific immune response process through the liver, thymus, glandular stomach and cecal tonsils. On the whole, comparing with the tissue numbers with significant differences of miR-155 between Dex+ND group and ND group, although *CTLA-4* had relative fewer tissues with significant differences, especially on 5dpi, the common differential tissues of miR-155 and CTLA-4 were mostly overlapped (such as heart and bursa on 2dpi; liver, spleen, thymus and cecal tonsil on 5dpi; liver, thymus and glandular stomach on 21dpi), suggesting that the immune regulatory function of *CTLA-4* was closely related to miR-155. In addition, in the Dex+ND group, *CTLA-4* showed a upward expression trend in the liver, bursa of *Fabricius* and glandular stomach from 2dpi to 21dpi, and reached the peak level on 5dpi in the cecal tonsil, and down-regulated and up-regulated in spleen and thymus respectively on 21dpi, further suggesting that the functions of *CTLA-4* participating in Dex affecting NDV immune response were different in different tissues and time points.

Interestingly, miR-155 and *CTLA-4* showed obvious game regulation relationship in the bursa of *Fabricius* and thymus on 2dpi, heart, thymus, spleen, bursa of *Fabricius* and glandular stomach on 5dpi, and liver and thymus on 21dpi, it further indicated that the miR-155-CTLA-4 pathway played a key regulatory role in the process of Dex affecting NDV immune response. Moreover, miR-155-CTLA-4 axis might be mainly involved in Dex affecting NDV immune response through bursa of *Fabricius*, thymus and liver, *etc*.

In conclusion, miR-155 and *CTLA-4* were the key factors involved in Dex-induced immunosuppression and NDV vaccine immune response, and 2dpi, 5dpi and 21dpi were the possible key regulatory time points. Moreover, the miR-155-CTLA-4 pathway in bursa of *Fabricius*, thymus and liver was one of the possible key mechanisms by which they were involved in the regulation of immune function. This study could lay the foundation for further research on the molecular mechanism of stress-induced immunosuppression affecting immune responses.

##  Supplemental Information

10.7717/peerj.14529/supp-1 Supplemental Information 1 CTLA-4 Raw data

10.7717/peerj.14529/supp-2 Supplemental Information 2 Raw Data

10.7717/peerj.14529/supp-3 Supplemental Information 3 Author checklist

## Additional Information and Declarations

### Data Availability

The following information was supplied regarding data availability: The raw data is available in the Supplemental Files and at GenBank: MW654205.
